# Association between Maternal *MTHFR* Polymorphisms and Nonsyndromic Cleft Lip with or without Cleft Palate in Offspring, A Meta-Analysis Based on 15 Case-Control Studies

**DOI:** 10.22074/ijfs.2015.4186

**Published:** 2015-02-07

**Authors:** Xinjuan Pan, Ping Wang, Xinjuan Yin, Xiaozhuan Liu, Di Li, Xing Li, Yongchao Wang, Hongle Li, Zengli Yu

**Affiliations:** 1 College of Public Health, Zhengzhou University, Zhengzhou, China; 2 Medical College, Henan University of Science and Technology, Luoyang, China

**Keywords:** Methylenetetrahydrofolate Reductase, Cleft Lip, Meta-Analysis

## Abstract

**Background:**

The methylenetetrahydrofolate reductase (MTHFR) is thought to be involved in the development of nonsyndromic cleft lip with or without cleft palate (NSCL/P). However, conflicting results have been obtained when evaluating the association between maternal *MTHFR* C677T and A1298C polymorphisms and the risk of NSCL/P. In light of this gap, a meta-analysis of all eligible case-control studies was conducted in the present study.

**Materials and Methods:**

A total of 15 case-control studies were ultimately identified after a comprehensive literature search and Hardy-Weinberg equilibrium (HWE) examination. Cochrane’s Q test and index of heterogeneity (I^2^) indicated no obvious heterogeneity among studies.

**Results:**

Fixed or random-effects models were used to calculate the pooled odds ratios (ORs). The results showed that the TT genotype in mothers increased the likelihood of having NSCL/P offspring 1.25 times (95% CI: 1.047-1.494) more than the CC homozygotes. Meanwhile, maternal TT genotype increased the risk of producing NSCL/P offspring in recessive model (OR=1.325, 95% CI: 1.124-1.562). However, the CT heterozygote and the CT+TT dominant models had no association with NSCL/P offspring compared with the CC wild-type homozygote model. Subgroup analyses based on ethnicity indicated that maternal TT genotype increased the likelihood of having NSCL/P offspring in Whites (OR=1.308, 95% CI: 1.059-1.617) and Asians (OR=1.726, 95% CI: 1.090-2.733) in recessive model. Also, subgroup analyses based on source of control showed that mothers with the 677TT genotype had a significantly increased susceptibility of having NSCL/P children in hospital based population (HB) when compared with CC homozygotes (OR=1.248, 95% CI: 1.024-1.520) and un- der the recessive model (OR=1.324, 95% CI: 1.104-1.588). Furthermore, maternal A1298C polymorphism had no significant association with producing NSCL/P offspring (dominant model OR=0.952, 95% CI: 0.816-1.111, recessive model OR=0.766, 95% CI: 0.567-1.036).

**Conclusion:**

MTHFR C677T polymorphism is associated with the risk of generating NSCL/P offspring, and being a 677TT homozygote is a risk factor. MTHFR A1298C polymorphism was not associated with generating NSCL/P offspring. However, further work should be performed to confirm these findings.

## Introduction

Cleft lip and palate is one of the most common congenital defects in humans (1), which is divided into two groups in genetics as syndromic and nonsyndromic (2). Of all patients with cleft lip and palate, only small portion are syndromic and most are nonsyndromic (3, 4). Based on clinical manifestations, nonsyndromic cleft lip and palate can be divided into nonsyndromic cleft lip with or without cleft palate (NSCL/P,OMIM 119530) and the palate only (CPO, OMIM 119540) (2). NSCL/P is a congenital facial malformation without any other structural or developmental abnormalities (1), and is different from CPO in embryologic origin and recurrence risks (5).

Although the etiology of NSCL/P is complex (6), numerous studies have reported that NSCL/P is associated with folate metabolism (7-10), and genes which encode key proteins of folate and methionine metabolism play a role in the susceptibility of NSCL/P (11). Thus, the gene encoding the methylenetetrahydrofolate reductase (*MTHFR*) enzyme is particularly attractive, because this enzyme is responsible for folate-dependent metabolism of homocysteine, which catalyses the reduction of 5, 10-methylenetetrahydrofolate to 5-methyltetrahydrofolate, the predominant circulatory form of folate and the carbon donor for the remethylation of homocysteine to methionine (12).

The gene encoding the MTHFR enzyme is known to have at least two functional polymorphisms namely, C677T (rs1801133) and A1298C (rs1801131) for which their roles in the mechanisms of folate enzyme have been extensively investigated (12-16). The 677T allele results in an alanine to valine substitution at codon 222 (A222V), resulting in a thermolabile enzyme with 70% reduction in specific catalytic activity (13, 14). Similarly, the 1298C allele results in a glutamic acid to alanine substitution at codon 429 (G429A), resulting in a 40% reduction of MTHFR activity *in vivo* (15, 16). The low MTHFR activity caused by MTHFR polymorphisms maybe results in higher homocysteine or lower plasma folate levels, which both are associated with many diseases such as Down’s syndrome and neural tube defect (15, 17).

It has been hypothesized that NSCL/P may be associated with *MTHFR* which encodes a key protein in folate and methionine metabolism (11). Thus, *MTHFR* has been wildly studied to examine the relationship between its polymorphisms and the risk of NSCL/P but conflicting results were reported. Some studies found that the *MTHFR* C677T variant is associated with NSCL/P (18, 19). However, other studies, carried out in various populations around the world, found no or variable association (7, 20-24). Some studies indicated that the genotype of infants at C677T made a major contribution to the occurrence of NSCL/P (25-27) but others did not (28-31). Some investigations showed that the maternal genotype for the *MTHFR* C677T polymorphism had a significant impact on the occurrence of NSCL/P in their offspring (25, 32-37), but, other studies did not support this finding (29, 38-41).

Similarly, studies on the *MTHFR* A1298C polymorphism also yielded inconsistent results. The effect of *MTHFR* A1298C polymorphism was diverse from being a risk factor (18, 33) to no risk at all (7, 19, 23). Neither infant (26, 29, 42) nor maternal (40) *MTHFR* A1298C polymorphism obtained positive association with NSCL/P risk. In light of this gap, a meta-analysis on infant *MTHFR* polymorphisms and NSCL/P susceptibility was performed by Pan et al. (43), which suggested that infant *MTHFR* C677T polymorphism was involved in the development of NSCL/P. However, whether maternal *MTHFR* polymorphisms related to having NSCL/P children is yet to be confirmed. To resolve this confusion, we did a meta-analysis focusing on maternal *MTHFR* polymorphisms and the risk of having offspring with NSCL/P.

## Materials and Methods

### Study question

Are maternal *MTHFR* polymorphisms (C677T or A1298C) risk factors for having a child with NSCL/P?

### Criteria for included studies

1. Explored the association of maternal *MTHFR* polymorphisms and NSCL/P children. 2. Casecontrol study design; considering the heterogeneity in different study designs, we only focused on casecontrol studies. Cross-sectional studies, case-parent triads, and transmission disequilibrium tests (TDT) designed studies were not included. 3. Cases were mothers who have children with NSCL/P. Control group were mothers without NSCL/P children and selected from the general population or hospital based population. 4. Provided distributions of the maternal *MTHFR* C677T and/or A1298C genotypes. 5. Control groups in studies did not deviate from Hardy-Weinberg equilibrium (HWE).

We did not consider the genotyping method. We only included information available from the publications and did not seek additional information by contacting primary authors. Studies were excluded if the disease was defined as either familial NSCL/P or orofacial clefts. Case reports, letters and review articles were excluded from the study.

### Strategies for identified studies

A comprehensive literature search was performed using PubMed, Springer, Elsevier Digital Dissertations Databases, Scopus, and ISI web of knowledge with MeSH terms retrieval and free words retrieval for relevant articles published in English up to 15^th^ March 2013. MeSH terms included "cleft lip" and "methylenetetrahydrofolate reductase (nadph2)". Free words included "cleft lip", "cleft", "lip", "methylenetetrahydrofolate" and "MTHFR". We extended our search to review the reference lists of retrieved articles and performed manual searching as a supplement. When a study had duplicate publication, only the most inclusive publication was considered. The full texts of candidate articles were examined by two investigators independently.

### Data extraction

Data from each eligible study were extracted on custom-made data collection forms by two authors independently. For inconsistent evaluations, agreements were reached following discussion in our study group. For each study, the following characteristics were collected: first author, publication year, country, source of controls, characteristics of study population (ethnicity, sample size of case and control), and distributions of the maternal *MTHFR* C677T or A1298C genotypes among cases and controls. For studies with multiple gene polymorphisms, only data concerning *MTHFR* polymorphisms were included in the analysis. No modification of original data was performed. In addition, HWE was calculated based on the genotypes among controls. The data were extracted from published manuscripts, thus no research ethics board approval was necessary.

### Statistical analysis

Stata 8.0 (Stata Corporation, College Station, TX) was used to perform all statistical analyses. Goodness-of-fit chi-squared test was used to assess the frequencies of *MTHFR* C677T and A1298C polymorphisms from expectation under HWE in controls. The strength of the association between the polymorphisms and NSCL/P was measured by ORs with 95% confidence intervals (CIs). The statistical significance of the observed OR was tested by Z-test. For the C677T polymorphism, we firstly used wild-type CC genotype as the reference group to evaluate the effects of the CT and TT genotypes on NSCL/P susceptibility. Then we estimated the effects of CT/TT versus CC and TT versus CT/CC assuming the dominant and recessive models of the T allele. Same procedures were also performed on the A1298C polymorphism. Furthermore, subgroup analyses were performed based on source of controls and ethnicity. Heterogeneity assumption between studies was examined by the Chi-square based on Q-test and I-square value (44). A ﬁxedeffect model using the Mantel–Haenszel method was selected to pool data if there was homogeneity (p>0.05), otherwise, the random-effects model, using the Der Simonian and Laird method, was conducted.

Sensitivity analyses using the step by step exclusion method were conducted to assess whether each individual study affected the final results. Publication bias was evaluated by Begg’s test, the Egger’s asymmetry test and visual inspection of funnel plots. All the P values were for a twosided test and p<0.05 was considered statistically signiﬁcant.

Sensitivity analyses using the step by step exclusion method were conducted to assess whether each individual study affected the final results. Publication bias was evaluated by Begg’s test, the Egger’s asymmetry test and visual inspection of funnel plots. All the P values were for a twosided test and p<0.05 was considered statistically signiﬁcant.

## Results

### Description of studies

**Fig 1:**
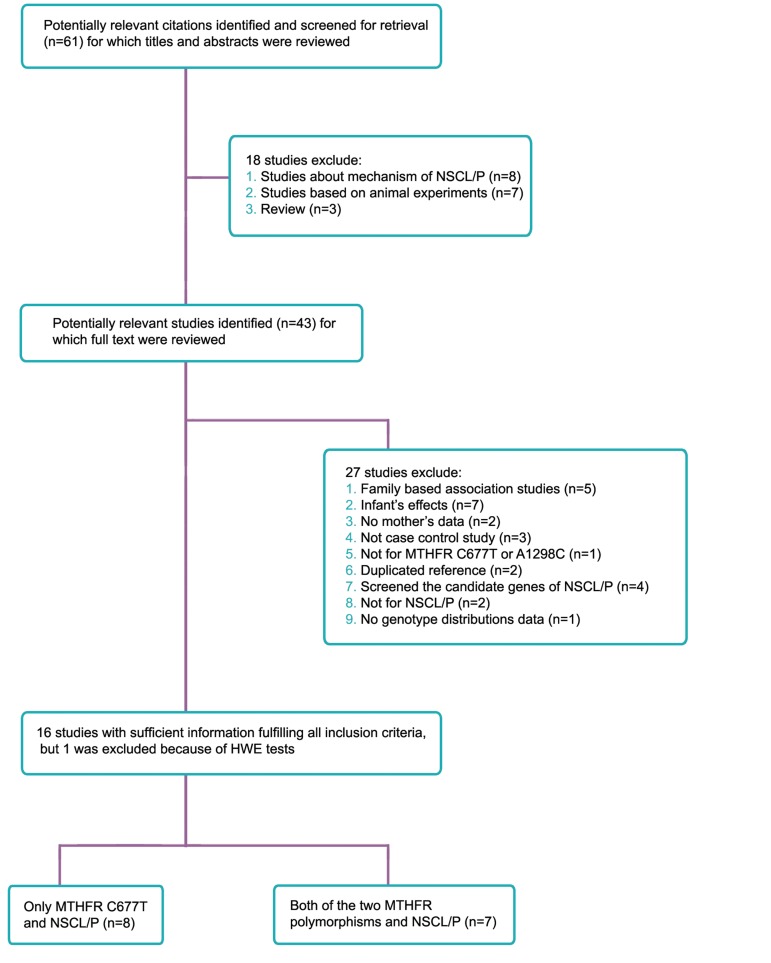
A flow diagram for selection of studies and specific reasons for exclusion in this meta-analysis. NSCL/P; Nonsyndromic cleft lip with or without cleft palate, HWE; Hardy-Weinberg equilibrium and MTHFR; Methylenetetrahydrofolate reductase.

Fifteen case-control studies were identified via our search strategy, for which 8 were concerned with C677T exclusively, while 7 studies had analyzed both variants (Fig 1).

The characteristic information of the included studies such as first author, publication year, country, ethnicity, source of controls, sample size (case/control), genotype distributions (case/ control) are presented in table 1 and table 2. Little’s study was excluded because the genotype distribution among the control group was not in HWE (p<0.05) (45).

**Table 1:** Main characteristics of MTHFR C677T polymorphism studies included in the meta-analysis


					Case	Control	Genotype (case/control)			Allele (case/control)		HWE
First author	Year	Country	Ethnicity	Source of	n	n	CC	CT	TT	C n (%)	T n (%)	(P)^##^

**Brandalizeet al. (21)**	2007	Brazil	White	HB	110	100	44/38	45/52	21/10	133(60)/128(64)	87(40)/72(36)	0.20
**Littleet al. (45)^a^**	2008	England	White	PB	96	226	46/86	42/119	8/21	134(70)/291(64)^#^	58(30)/161(36)	0.03
**Guo et al.(22)**	2009	China	Asian	HB	97	102	26/20	49/57	22/25	101(52)/97(48)	93(48)/107(52)	0.22
**Wanget al. (35)**	2012	China	Asian	HB	89	64	10/15	41/39	38/10	61(34)/69(54)	117(66)/59(46)	0.07
**Chornaet al. (25)**	2011	Ukraine	European	PB	27	50	19/34	2/2	6/14	40(74)/70(70)	14(26)/30(30)	0.05
**Ali et al.(19)**	2009	India	Asian	PB	116	214	78/176	37/36	1/2	193(83)/388(91)	39(17)/40(9)	0.92
**Bufalinoet al. (36)**	2010	Brazil	Mixed	HB	106	184	49/95	50/72	7/17	148(70)/262(71)	64(30)/106(29)	0.53
**Gasparet al. (11)**	2004	Brazil	Mixed	HB	336	644	174/327	131/269	31/48	479(71)/923(72)	193(29)/365(28)	0.47
			Whites	HB	235	474	126/235	88/202	21/37	340(72)/672(71)^#^	130(28)/276(29)^#^	0.48
			Nonwhites	HB	77	90	40/43	31/39	6/8	111(72)/125(69)^#^	43(28)/55(31)^#^	0.84
			Unclassified	HB	24	80	8/49	12/28	4/3	28(58)/126(79)^#^	20(42)/34(21)^#^	0.68
**Gasparet al. (46)**	1999	Brazil	White	HB	59	90	30/37	19/40	10/13	79 (67)/114 (63)	39 (33)/66 (37)	0.68
**Millset al. (37)**	2008	Ireland	White	HB	465	1599	205/715	212/721	48/163	622(67)/2151(67)^#^	308(33)/1047(33)^#^	0.34
**Mostowskaet al. (47)**	2006	Poland	European	PB	121	81	60/42^*^	46/33^*^	15/6^*^	166(69)/117(72)	76 (31)/45 (28)	0.89
**Pezzettiet al. (34)**	2004	Italy	White	HB	104	289	27/95	47/151	30/43	101(49)/341(59)	107(51)/237(41)	0.17
**Shotelersuket al. (33)**	2003	Thailand	Asian	PB	67	202	46/154	19/46	2/2	111(83)/354(88)	23(17)/50(12)	0.48
**Sozenet al. (23)**	2009	Venezuela	Mixed	PB	168	138	109/66	49/65	10/7	267(79)/197(71)	69(21)/79(29)	0.07
**Van Rooijet al. (7)**	2003	Netherlands	White	PB	148	170	78/84	55/74	15/12	211(71)/242(71)^#^	85(28)/98(29)^#^	0.43
**Tolarovaet al. (48)**	1998	Argentina	White	PB	93	84	39/39	37/33	17/12	115(62)/111(66)^#^	71(38)/57(34)^#^	0.26


PB; Population based, HB; Hospital based, HWE; Hardye Weinberg equilibrium, NA; Not available, a; Not enter final analysis because not fit HWE, *; Numbers calculated by text describe, #; Numbers calculated by the distribution of genotype and ##; P value of HWE were calculated by original data.

**Table 2:** Main characteristics of MTHFR A1298C polymorphism studies included in the meta-analysis


					Case	Control	Genotype (case/control)			Allele (case/control)		HWE
Firstauthor	Year	Country	Ethnicity	Source ofcontrols	n	n	AA	AC	CC	A n (%)	C n (%)	(P)^##^

**Van Rooijet al. (7)**	2003	Netherlands	White	PB	125	159	57/76	52/67	16/16	166(66)/219(69)^#^	84(34)/99(31)^#^	0.83
**Shotelersuket al. (33)**	2003	Thailand	Asian	PB	67	202	30/108	33/80	4/14	93(69)/296(73)	41(31)/108(27)	0.88
**Pezzettiet al. (34)**	2004	Italy	White	HB	104	254	57/121	36/130	11/38	150(72)/372(64)	58(28)/206(36)	0.74
**Mills et al.(37)**	2008	Ireland	White	HB	366	1050	179/519	164/439	23/92	522(71)/1477(70)^#^	210(29)/623(30)^#^	0.95
**Ali et al.(19)**	2009	India	Asian	PB	116	214	64/99	47/97	5/18	175(75)/295(69)	57(25)/133(31)	0.4
**Sozen et al.(23)**	2009	Venezuela	Mixed	PB	168	138	119/101	47 /33	2 /4	285(85)/235(85)	51(15)/41(15)	0.52
**Tolarovaet al. (48)**	1998	Argentina	White	PB	86	78	56/50	27/25	3/3	114(78)/125(80)^#^	33(22)/31(20)^#^	0.95


PB; Population based, HB; Hospital based, HWE; Hardye Weinberg equilibrium, NA; Not available, #; Numbers calculated by the distribution of genotype and ##; P value of HWE were calculated by original data.

### Heterogeneity test

**Fig 2:**
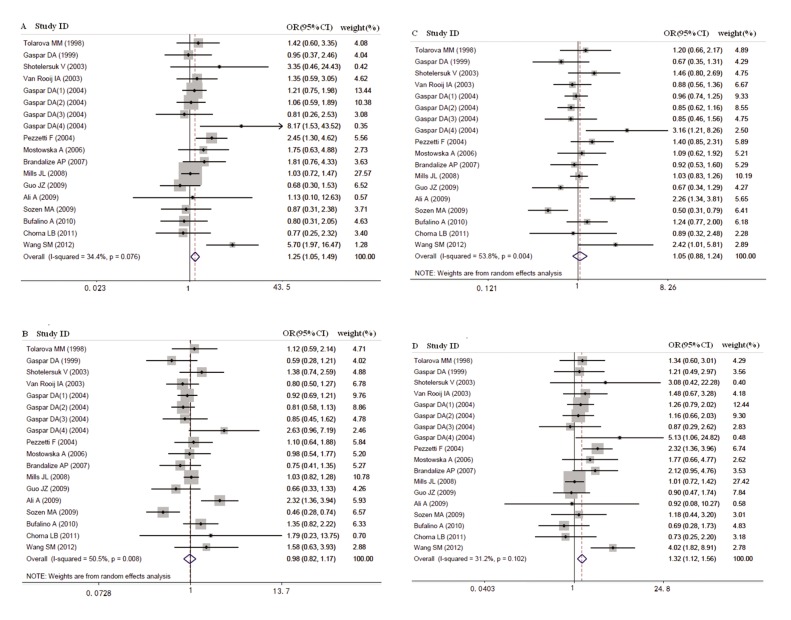
Forest plots of association between MTHFR C677T polymorphism and NSCLP risk. A; TT vs. CC, B; CT vs. CC, C; CT+TT vs. CC and D; TT vs. CT+CC.

**Fig 3:**
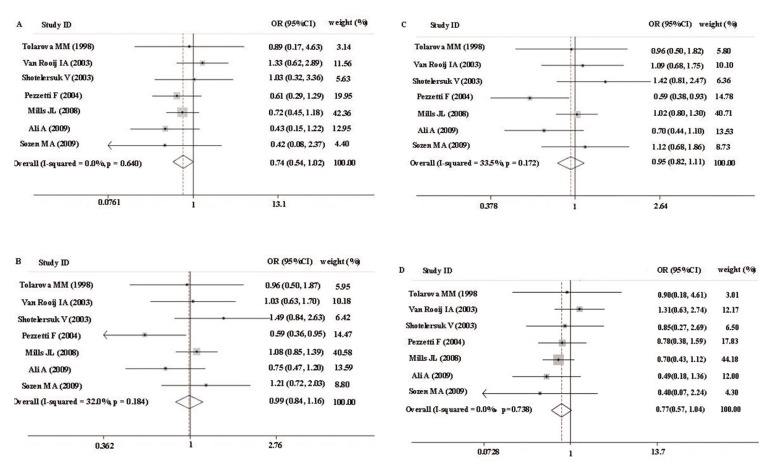
Forest plots of association between MTHFR A1298C polymorphism and NSCLP risk. A; CC vs. AA, B; AC vs. AA, C; AC+CC vs AA and D; CC vs. AC+AA.

Overall, for maternal *MTHFR* C677T polymorphism, there were significant heterogeneity for heterozygote comparison (CT versus CC: P_h_=0.008) and dominant model comparison (CT+TT versus CC: P_h_=0.004), but not for the homozygote comparison (TT versus CC: P_h_=0.076) and the recessive model comparison (TT versus CT+CC: P_h_=0.102) (Table 3, Fig 2). Thus, we performed subgroup analysis stratified by source of controls and ethnicity to assess the cause of heterogeneity. Results suggested that ethnicity and source of controls were contributing to substantial heterogeneity. In A1298C polymorphism studies, there were no significant heterogeneity for all comparisons (all P_h_>0.05, Table 4, Fig 3).

### The synthesis of effect size and subgroup analysis

Regarding maternal C677T polymorphism and NSCL/P offspring, 15 studies with a total of 2442 cases and 4655 controls were included. Maternal TT genotype contributed to elevated risks of having an NSCL/P child compared with the CC wild-type genotype (OR=1.251, 95% CI: 1.047- 1.494), and this effect appeared only in the HB population (OR=1.248, 95% CI: 1.024-1.520) after subgroup analysis by source of controls and ethnicity. Under the recessive model, 677TT conferred increased susceptibility to having a child with NSCL/P when using CT+CC as reference (OR=1.325, 95% CI: 1.124-1.562), and this effect appeared in Asians (OR=1.726, 95% CI: 1.090-2.733), Whites (OR=1.308, 95% CI: 1.059-1.617), and the HB population (OR=1.324, 95% CI: 1.104-1.588) after further subgroup analysis. However, the CT heterozygote and the CT+TT dominant model had no association with bearing NSCL/P offspring when compared with CC wild-type genotype (all 95% CI include 1) (Table 3, Fig 2).

With respect to the association between maternal *MTHFR* A1298C polymorphism and NSCL/P offspring, 7 studies with 1032 cases and 2095 controls were included. There was no association between the maternal *MTHFR* A1298C polymorphism and having NSCL/P offspring under all genetic models, since the null value of all ORs (null value=1) was well inside the 95% confidence intervals (Table 4, Fig 3).

### Sensitivity analyses and publication bias

No individual study affected the final results according to sensitivity analyses (data not shown). Funnel plots of all studies revealed no asymmetrical distribution of ORs (Figes4-5), suggesting no significant publication bias in this study. Begg’s test and Egger’s test provided further statistical evidence of no significant publication bias in this meta-analysis (all p>0.05) (Table 5).

**Table 3:** Results of Meta-analysis of MTHFR C677T polymorphism for studies on NSCL/P


Groups	Studynumber	Sample size(case/control)	TT versus CC			CT versus CC			CT+TT versus CC (dominant)			TT versus CT+CC (recessive)		
			OR(95% CI)	I^2^(%)	P_h_	OR (95% CI)	I^2^(%)	P_h_	OR (95% CI)	I^2^(%)	P_h_	OR(95% CI)	I^2^(%)	P_h_

**Overall**	15	2442/4655	1.251(1.047,1.494)	34.4	0.076	0.982(0.824,1.171)	50.5	0.008^#^	1.048(0.884, 1.244)	53.8	0.004^#^	1.325(1.124, 1.562)	31.2	0.102	
**Ethnicity**															
Asians	4	369/582	1.565(0.887,2.761)	71.1	0.016^#^	1.378(0.796, 2.385)	62.2	0.047^#^	1.505(0.849, 2.667)	67.5	0.026^#^	1.726(1.090, 2.733)	65.5	0.034^#^	
Whites	8^*^	1269/2853	1.243(0.992,1.558)	13.2	0.327	0.920(0.794, 1.066)	0.0	0.704	0.973(0.847, 1.118)	0	0.712	1.308(1.059, 1.617)	27.7	0.207	
Others	6^*^	804/1220	1.172(0.839,1.639)	26.1	0.238	0.969(0.666, 1.410)	66.6	0.010^#^	1.010(0.702, 1.452)	68	0.008^#^	1.194(0.865, 1.648)	1.9	0.404	
**Source of control**															
HB	8	1702/3716	1.248(1.024,1.520)	56.9	0.01^#^	0.957(0.821, 1.114)	17.3	0.279	1.026(0.861, 1.223)	38.4	0.093	1.324(1.104, 1.588)	55.3	0.013^#^	
PB	7	740/939	1.262(0.842,1.892)	0	0.842	1.052(0.670, 1.650)	73.0	0.001^#^	1.074 (0.723,1.596)	70.7	0.002^#^	1.329(0.899, 1.965)	0	0.885	


*; In Gaspar et al. (11) study, there have the data not only of whites but also of others, I^2^; Quantification of the heterogeneity, P_h_; P values for heterogeneity from Q test, #; Random-effect model was used when p value for heterogeneity test <0.05, otherwise, fix-effect model was used, PB; Population based and HB; Hospital based.

**Table 4:** Results of Meta-analysis of MTHFR A1298C polymorphism for studies on NSCL/P


Groups	Studynumber	Sample size(case/control)	TT versus CC			CT versus CC			CT+TT versus CC (dominant)			TT versus CT+CC (recessive)		
			OR(95% CI)	I^2^(%)	P_h_	OR (95% CI)	I^2^(%)	P_h_	OR (95% CI)	I^2^(%)	P_h_	OR(95% CI)	I^2^(%)	P_h_

**Overall**	7	1032/2095	0.744	0	0.64	0.991	32	0.184	0.952	33.5	0.172	0.766	0	0.738	
			(0.545,1.016)			(0.844,1.164)			(0.816,1.111)			(0.567,1.036)			
**Ethnicity**															
Asians	2	183/416	0.611	15.7	0.276	0.986	69.5	0.070	0.929	73.1	0.054	0.618	0	0.479	
			(0.281,1.329)			(0.687,1.413)			(0.655,1.316)			(0.289,1.321)			
Whites	4	681/1541	0.794	0	0.505	0.966	39.1	0.177	0.938	38	0.184	0.822	0	0.565	
			(0.561,1.125)			(0.797,1.170)			(0.780,1.126)			(0.588,1.150)			
Others	1	168/138	0.424	-	-	1.209	-	-	1.124	-	-	0.404	-	-	
			(0.076,2.365)			(0.720,2.029)			(0.680,1.858)			(0.073,2.237)			
**Source**															
**of control**															
HB	2	470/1304	0.690	0	0.715	0.828	79.3	0.028^#^	0.807	76.9	0.037^#^	0.722	0	0.797	
			(0.459,1.036)			(0.457,1.501)			(0.476,1.366)			(0.487,1.071)			
PB	5	562/791	0.834	0	0.448	1.038	0	0.444	1.008	5.9	0.373	0.839	0	0.526	
			(0.513,1.358)			(0.818,1.316)			(0.802,1.266)			(0.523,1.345)			


I^2^; Quantification of the heterogeneity, P_h_; P values for heterogeneity from Q test, #; Random-effect model was used when p value for heterogeneity test <0.05, otherwise, fix-effect model was used, " -"; no I^2^ and Ph because only 1 study in this subgroup, PB; Population based and HB; Hospital based.

**Fig 4:**
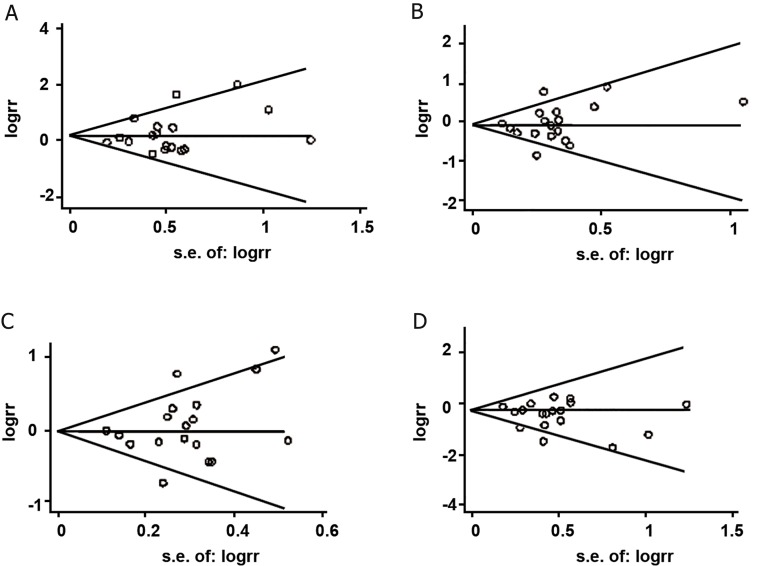
Funnel plots for included studies of MTHFR C677T polymorphism and NSCLP risk. Begg’s funnel plot with pseudo 95% confidence limits. A; TT vs. CC, B; CT vs. CC, C; CT+TT vs. CC ad D; TT vs. CT+CC.

**Fig 5:**
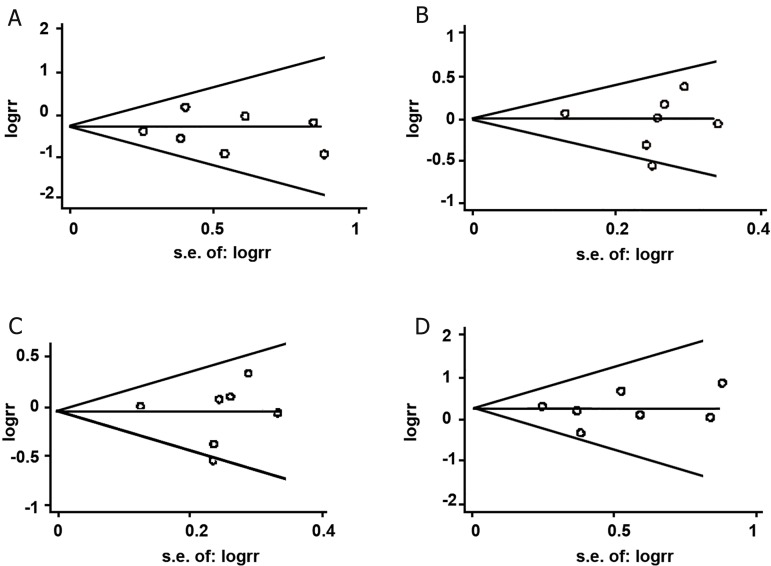
Funnel plots for included studies of MTHFR A1298C polymorphism and NSCLP risk. Begg’s funnel plot with pseudo 95% confidence limits. A; CC vs. AA, B; AC vs. AA, C; AC+CC vs. AA and D; CC vs. AC+AA.

**Table 5:** Results of Egger’s and Begg’s tests


Comparison	Egger’s test			Begg’s test	
	t	P	95% CI	Z	P

**C677T**
TT vs. CC	1.27	0.221	-0.566, 2.272	1.21	0.225
CT vs. CC	0.68	0.504	-1.117, 2.180	0.53	0.596
CT+TT vs. CC	1.04	0.315	-0.892, 2.603	0.83	0.405
TT vs. CT+CC	-0.98	0.340	-2.076, 0.760	0.23	0.820
**A1298C**					
CC vs. AA	-0.24	0.821	-2.444, 2.029	0.30	0.764
AC vs. AA	-0.34	0.750	-4.335, 3.332	0.30	0.764
CC+AC vs. AA	-0.14	0.891	-4.155, 3.716	0.60	0.548
CC vs. AC+AA	-0.30	0.774	-2.245, 1.771	0.00	1.000
					


## Discussion

### Principal findings

In the present meta-analysis, we focused on the relationship of maternal *MTHFR* polymorphisms (C677T and A1298C) and risk of having NSCL/P offspring. Results showed that mothers carrying the 677TT genotype had an increased susceptibility to have an NSCL/P offspring under both the recessive model and the CC homozygote model, but the CT heterozygote and the CT+TT dominant models had no association with bearing NSCL/P offspring when compared with CC genotype. This suggests that only the maternal 677TT genotype is a risk factor for having NSCL/P offspring. Subgroup analyses based on ethnicity and source of control showed that under the recessive model, maternal 677TT genotype increased the risk of having NSCL/P offspring in Whites, Asians, and the HB population, providing further evidence that the TT genotype is the risk factor. However, maternal *MTHFR* A1298C polymorphism had no association with having NSCL/P offspring under all genetic models, and results did not differ when subgroup analyses were undertaken.

In the present study, results from the dominant model were consistent with that reported by Verkleij-Hagoort et al. (49), which used single dominant model and also indicated that the maternal *MTHFR* C677T and A1298C polymorphisms were not independently associated with CLP with pooled ORs of 1.2 (95% CI: 0.9-1.5) and 1.0 (95% CI: 0.7-1.2). Results from the codominant model were similar to the study by Luo et al. (50), which only used the co-dominant model and also revealed that the maternal 677TT genotype elevated the risk of CL/P with a pooled OR of 1.32 (95% CI: 1.06-1.63) when compared with the normal 677CC genotype. However, the results of subgroup analyses were different. Luo et al. (50) demonstrated that maternal 677TT genotype increased the risk of having CL/P offspring in the white population with an OR of 1.36 (95% CI:1.05-1.76), while in present subgroup analyses, based on the same factors and the same genetic model, the effect was found in the hospital based population but not in Whites or Asians.

There are several reasons for the inconsistency between the present study and the other two meta-analyses. First, the applied genetic models are different. It is notable that besides the co-dominant and dominant models, the recessive model was also applied in our present meta-analyses and the results further supported the view that maternal 677TT genotype is a risk factor and is associated with having NSCL/P offspring. Second, types of study designs are different. Studies included in the two meta-analysis covered case-control and cohort studies (51) whereas our study was restricted to case-control studies. Third, the number of included studies varied. There were 10 and 12 studies included in the 2007 and 2012 metaanalyses respectively, but our meta-analysis comprised of 15 studies.

Furthermore, Verkleij-Hagoort et al. (49) and Luo et al. (50) had estimated infant *MTHFR* polymorphisms and CL/P susceptibility under the dominant and co-dominant models respectively in their meta-analysis, and all pooled results revealed no statistical association between infant C677T and A1298C variants and risk of CL/P. While meta-analysis performed by Pan et al. (43) which combined co-dominant, dominant and recessive models suggested that the infant 677TT genotype is associated with CL/P in recessive model. However, we did not examine infant *MTHFR* polymorphisms in the present study, so we can not conclude which one (maternal or infant *MTHFR* polymorphisms) is more related and more important with regards to NSCL/P.

### Strength and weakness of the review

The associations between NSCL/P susceptibility and maternal *MTHFR* polymorphisms have been widely examined but provided inconsistent results. This absence of consensus among individual studies might be due to different ethnicities and study populations, different laboratory procedures used for genotyping, variable environment and diet, selection bias from both cases and controls subjects, and insufficient sample size (25, 33, 35, 36, 38, 39). However, meta-analyses have the advantage of increased statistical power by pooling the results from small individual studies and also can examination the variability between studies.

In the present study, to ensure the representativeness of the study subjects, we performed HWE test on the control groups in the final selected studies. Also, only NSCL/P was included to reduce etiologic heterogeneity. Furthermore, subgroup analyses based on ethnicity and source of control were performed to reduce ethnic heterogeneity and selection bias. However, certain limitations also need to be acknowledged. For one thing, the information of environmental interventions or maternal periconceptional behaviors was not available in our study, whereas these factors may influence the whole pregnancy outcome. For another thing, heterogeneity present in overall or subgroup analyses might be due to different regions, various genotyping methods and others mentioned above. But we only separated study populations into three groups: Asians, Whites, and others, and we did not perform any subgroup analysis based on genotyping method. Last, but not least, only case-control studies were selected, which are less powerful for genetic associations than transmission disequilibrium test in familybased designs.

### Possible mechanism

It is not possible to explain the mechanism of the association between NSCL/P susceptibility and maternal *MTHFR* polymorphisms in the present study. However, the deficiency of nutritional folic acid during embryonic development has been proposed as a factor in the etiology of NSCL/P, and many studies suggest that maternal ingestion of folic acid during early pregnancy can reduce the risk of NSCL/P (7-10). According to this evidence, it has been hypothesized that NSCL/P might be associated with folate metabolism and polymorphic variants of the genes which encode key proteins in folate and methionine metabolism may play a role in the susceptibility of NSCL/P (11).

MTHFR is an important enzyme in folate metabolism. Normal MTHFR activity is crucial to maintain the pool of circulating folate and methionine and to prevent the accumulation of homocysteine (12). C677T and A1298C are two common functional single nucleotide polymorphisms localized in *MTHFR*, and their roles in the mechanisms of folate enzyme have been extensively researched. Compared to the CC and CT genotypes, the TT genotype is characterized by 70% enzymatic activity reduction (52). And it is the reason that the concentrations of folate in serum, plasma, and red blood cells decrease and plasma homocysteine concentrations increase mildly (12-14). Similarly, the A1298C polymorphism, resulting in a glutamic acid to alanine substitution at codon 429, also causes decreasing MTHFR enzyme activity, but is not associated with higher homocysteine or lower plasma folate levels (15, 16). This mechanism can explain all the results we gained in present study.

In addition, it is worth noting that linkage disequilibrium (LD) exists between *MTHFR* C677T and A1298C. Some studies had reported a stronger MTHFR activity loss in combined heterozygote (C677T/A1298C) in neural tube defect cases (15, 53), however, there are few studies that investigated the relationship between this compound genotype and NSCL/P risk, and quantitative analysis cannot be conducted owing to incomplete data.

## Conclusion

The results of this study give supporting evidence for significant association between maternal *MTHFR* C677T polymorphism and risk of having an NSCL/P offspring, and confirm mothers with the 677TT genotype are susceptible to have a child with NSCL/P. Nevertheless, in view of the limitations of the present study, our findings should be interpreted prudently. Further work, especially those that address the combined effects of genetic and environmental factors on a specific phenotype of orofacial clefts should be performed to evaluate these findings.
